# Evaluation of the intraoperative specimens of the thoracic and abdominal aorta

**DOI:** 10.1186/1749-8090-8-110

**Published:** 2013-04-24

**Authors:** Andrzej Juraszek, Günther Bayer, Tomasz Dziodzio, Artur Kral, Günther Laufer, Marek Ehrlich

**Affiliations:** 1 Department of Cardiac Surgery, Medical University of Vienna, Währinger Gürtel 18-20, Vienna, 1090, Austria; 2 Department of Pathology, Medical University of Vienna, Vienna, Austria

## Abstract

**Background:**

Little is known about the histological patterns of acute and chronic aortic pathology with regard to medial degeneration, atherosclerosis and aortitis as well as their distribution in different age groups. The aim of the study was to evaluate histopathological findings of intraoperatively gained aortic specimens with regard to the incidence of medial degeneration, atherosclerosis and aortitis.

**Methods:**

Intraoperatively gained aortic specimens were evaluated in 151 patients including 83 (55%) aortic aneurysms (65 thoracic, 18 abdominal) and 68 (45%) acute type A aortic dissections. Histological stainings used were hematoxylin and eosin, Van Gieson as well as alcian blue. Patients were stratified according to above and below 65 years of age. High grade medial degeneration represented pooling of mucoid material in the whole aortic wall. High grade atherosclerosis represented severe intimal fibrosis, massive accumulation of macrophages and foam cells or massive calcification of the aortic wall.

**Results:**

Medial degeneration was diagnosed in 106 (70%) patients including 55 (52%) aortic aneurysms and 51 (48%) acute type A aortic dissections. High grade medial degeneration was found in 50% of patients with thoracic aortic aneurysms < 65 years of age vs. 44% in patients ≥ 65 years of age (p = 0.64) and in 36% of patients with thoracic aortic dissections < 65 years of age vs. 14% in patients ≥ 65 years of age (p = 0.07). Atherosclerosis was diagnosed in 71 (47%) patients including 46 (65%) aortic aneurysms and 25 (35%) aortic dissections. High grade atherosclerosis was found in 23% of patients with thoracic aneurysms < 65 years of age vs. 36% in patients ≥ 65 years of age (p = 0.24) and in 13% of patients with aortic dissections < 65 years of age vs. 52% in patients ≥ 65 years of age (p < 0.001). Aortitis was rare (n = 2).

**Conclusions:**

Medial degeneration was the most frequent diagnosis in this series of aortic specimens. Medial degeneration was equally common in patients above and below 65 years of age. However in cases with acute type A aortic dissections, high grade atherosclerosis was the leading histopathological diagnosis in patients older than 65 years. Acute type A aortic dissections seem to have different underlying pathologies in different age groups.

## Background

Diagnostic as well as treatment algorithms in acute and chronic aortic pathology are diameter oriented [1-3]. Recently, a more thorough understanding of the underlying mechanism by which this disease develops has changed the way of interpreting morphological findings by imaging [4-6]. However, little is known about the histological patterns of acute and chronic aortic pathology with regard to medial degeneration, atherosclerosis and aortitis as well as their distribution in different age groups. While medial degeneration is reported to be the leading histologic finding in cases of aneurysm and dissection, the role of atherosclerosis and inflammatory processes seems to be underestimated [7]. Additionally, the mean age of patients operated due to aortic pathology, significantly increased in the last few years. This fact created a new patients’ cohort probably with pluricausal pathology of the aorta including medial degeneration, atherosclerosis and inflammatory processes [8].

The aim of this study was to evaluate histopathological findings of intraoperatively gained aortic specimens with regard to the incidence of medial degeneration and atherosclerosis. The study hypothesis was that various aortic pathologies show different histopathological patters in patients of different age groups.

## Methods

One hundred and fifty-one intraoperatively gained specimens of the entire aorta were gained at our institution (30% female). Mean age of patients was 58 ±15 years. Fifty-six patients (37%) were older than 65 years of age. Thoracoabdominal aneurysms were excluded from this analysis due to their multisegmentality. Patients were stratified according to above and below 65 years of age.

### Clinical risk factors and diagnoses

The presence of hypertension, diabetes mellitus, dyslipidemia, family history of the aortic disease, connective tissue disease and previous inflammatory process were registered. Previous inflammatory process included cases of aortititis, inflammatory process of the heart, systemic infection and systemic autoimmune disease.

### Staining

Hematoxylin-eosin (HE) staining was used routinely as a standard diagnostic tool. Van Gieson's staining was used to analyze elastic fibers. Finally, alcian blue staining was used to detect acid mucopolysaccharides. Magnifications of 40×, 100×, 200× and 400× were used.

### Medial degeneration

**Figure 1:**
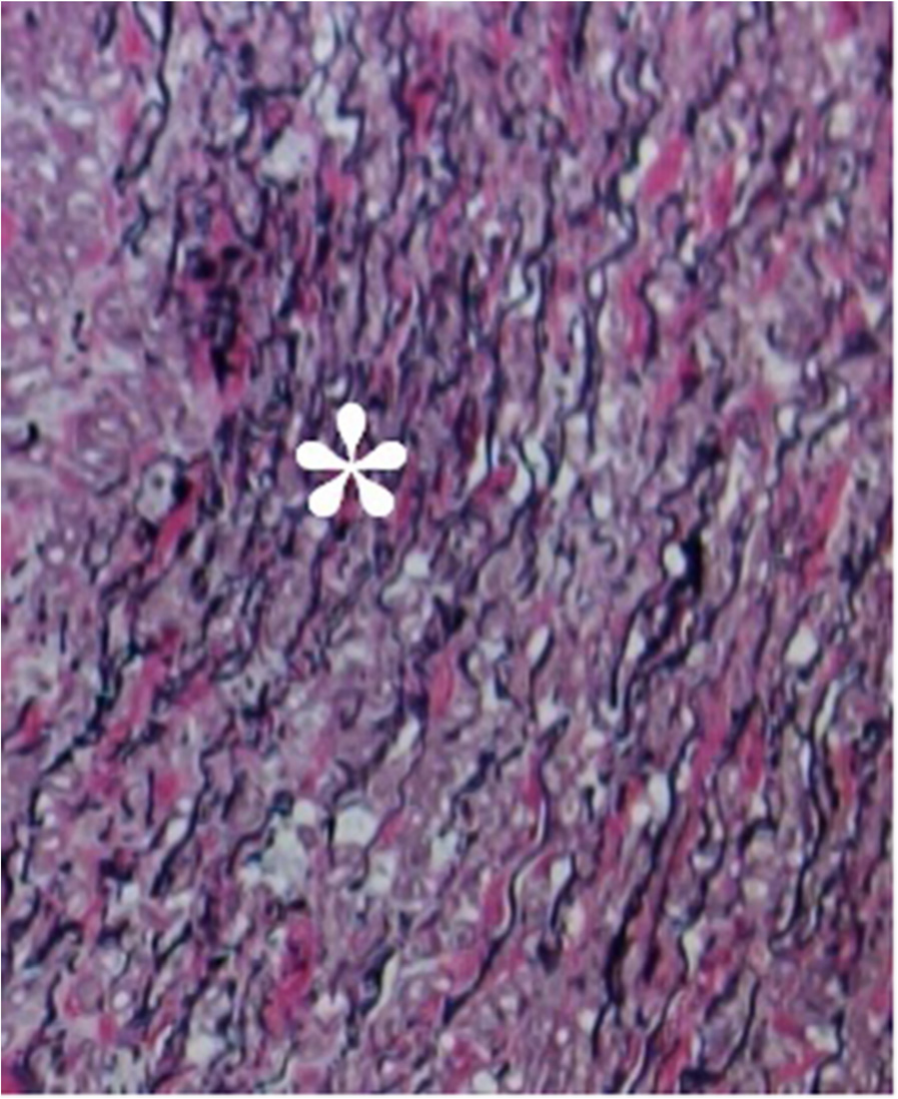
**Section of aortic wall with low grade medial degeneration.** Focal necking of elastic fibers (*). [Van Gieson's staining, original magnification 400×].

**Figure 2:**
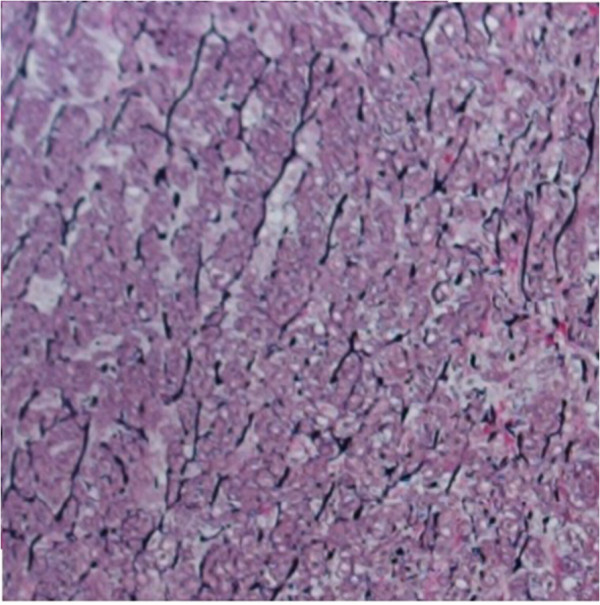
**Section of aortic wall with high grade medial degeneration.** Generalized loss of elastic fibers. [Van Gieson's staining, original magnification 400×].

**Figure 3:**
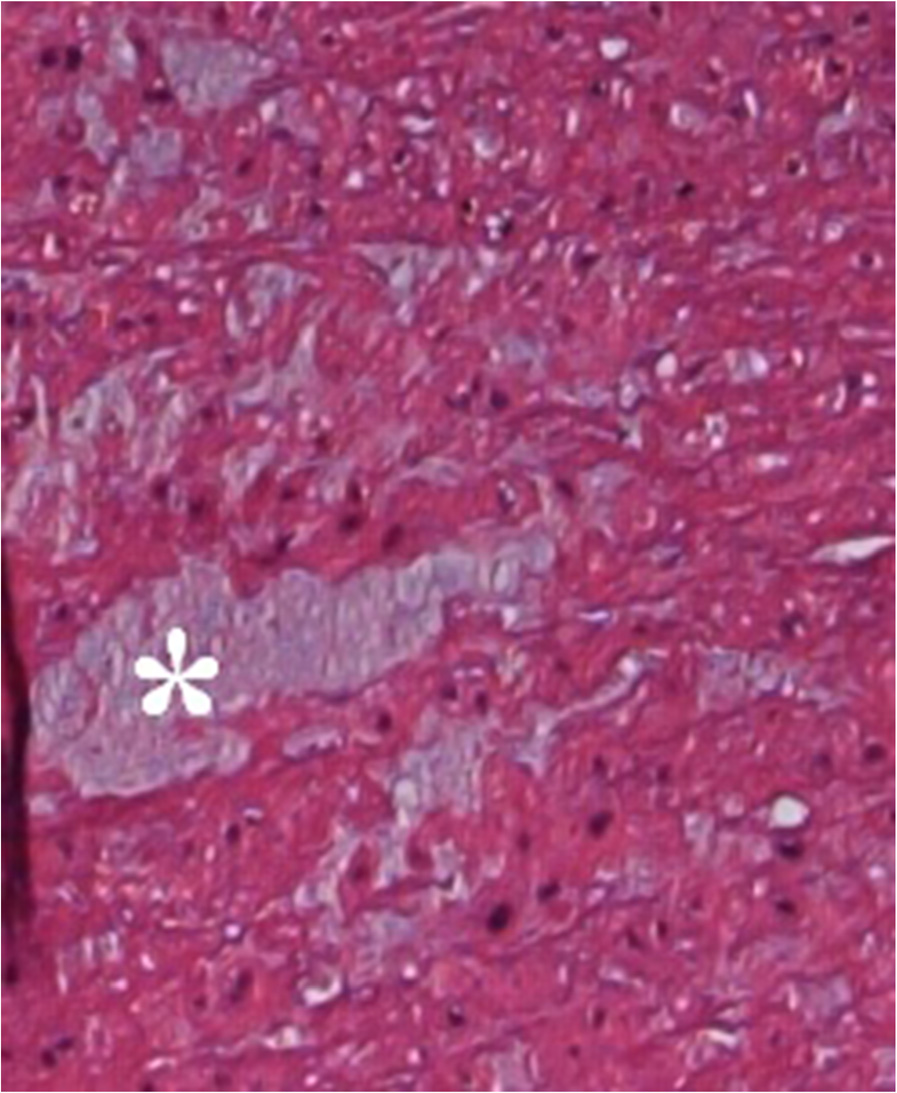
**Mucoid degeneration of the aorta.** Mucopolysaccharides are accumulated in so called « cysts » (*). [Hematoxylin-eosin staining, original magnification 400×].

In our study the diagnosis of medial degeneration was categorized into 2 grades, according to the severity of the pathology. Low grade medial degeneration included specimens with focal accumulation of alcian positive mucoid material with some fragmentation and disruption of the elastic frame (Figure 1). High grade medial degeneration represented disseminated mucoid degeneration with pooling of mucoid material in the whole aortic wall leading to a tiger skin pattern [9] (Figures 2 and 3).

### Atheroscerosis

**Figure 4:**
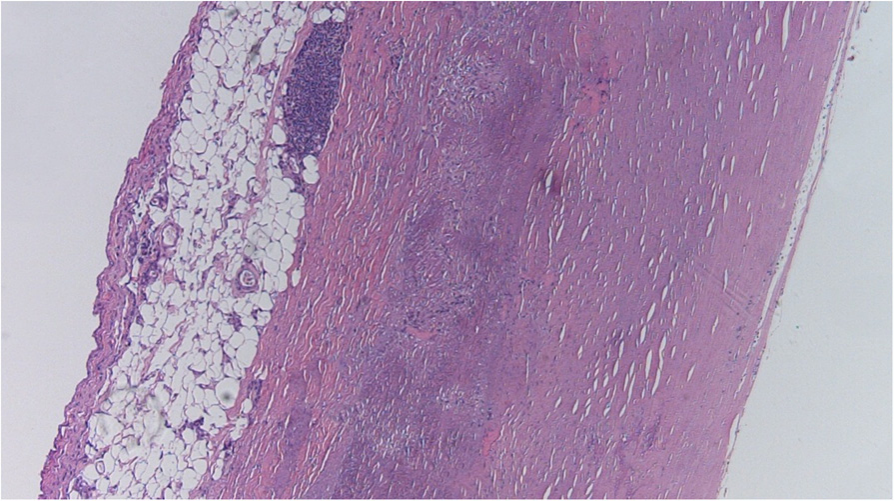
**Atherosclerosis, grade 1.** This mild form of atherosclerosis is caused by connective tissue proliferation in tunica intima also known as intimal fibrosis. [Hematoxylin-eosin staining, original magnification 40×].

**Figure 5:**
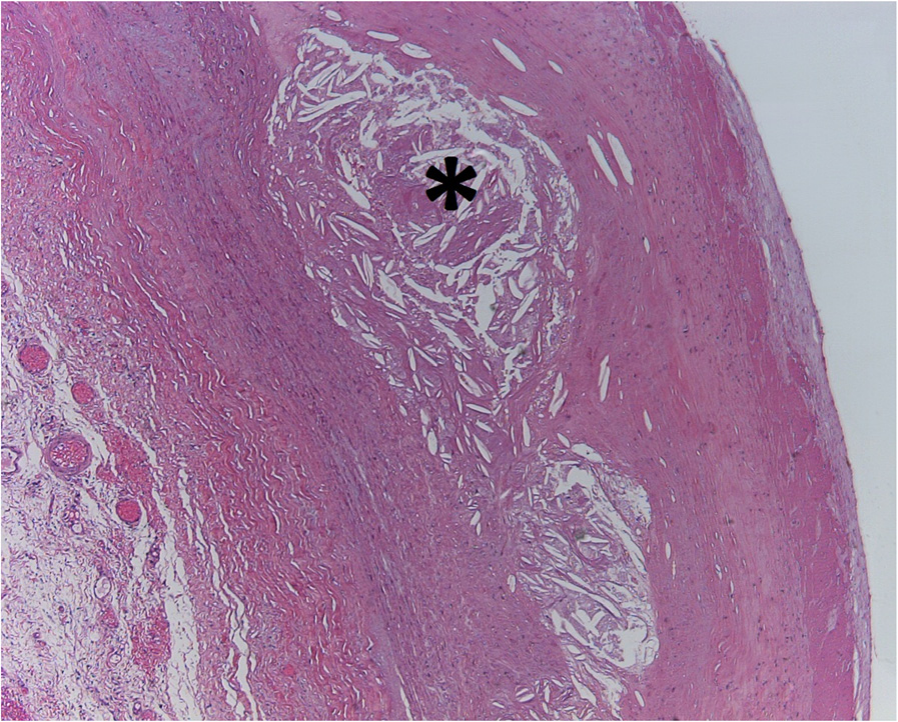
**High grade atherosclerosis.** Fibrosis of the tunica intima. Characteristic focal swelling in the aortic wall called atheroma consists of cholesterol cristals (*). [Hematoxylin-eosin staining, original magnification 200×].

Similarly to our classification of medial degeneration, the diagnosis of atherosclerosis was divided into 2 grades. Low grade atherosclerosis only included intimal fibrosis (Figure 4). High grade atherosclerosis represented massive intimal fibrosis, massive accumulation of macrophages and foam cells and in some cases massive calcification [9,10] (Figure 5). Additionally atherosclerosis was classified according to the American Heart Association [10].

### Aortitis

Due to the expected low number of cases with aortitis there was no classification necessary. Every case was described individually.

### Ethics approval

The study was conducted in the Department of Cardiac Surgery, Medical University of Vienna, Austria. An ethics approval has been granted by the Ethics Committee of the Medical University of Vienna. Due to the retrospective character of the study there was no informed consent needed to conduct the study.

### Statistics

Normally distributed continuous data are presented as mean and standard deviation. Categorical variables are presented as absolute and relative frequencies. Comparisons of continuous data were performed by Mann Whitney U tests and groups of categorical data were compared by Fisher tests. A two- sided p-value below 0.05 was considered as statistically significant. Calculations were performed with SPSS for Mac OsX (version 16.0; SPSS Inc, an IBM Company, Chicago, Ill).

A comparative analysis between age and sex groups has been performed. All of the patients were stratified according to above and below 65 years of age. Fisher test was used to compare the clinical diagnoses were with the histopathology results.

## Results and discussion

### Clinical diagnosis

Aortic specimens were collected in 151 patients including 83 (55%) aortic aneurysms and 68 thoracic aortic dissections (TAD). The aneurysmal group included 65 (78%) thoracic (TAA) and 18 (22%) abdominal cases (AAA). There were 63 chronic ascending aortic aneurysms, one case of an acute rupture of the ascending aorta and one case of a chronic aneurysm of the aortic arch. All cases with AAA were chronic. The group with TADs included 59 (87%) acute dissections and 9 (13%) chronic cases. The data concerning hypertension, diabetes mellitus, dyslipidemia, family history of the aortic disease, connective tissue disease and previous inflammatory process are presented in (Tables 1, 2 and 3). The patients with an AAA showed a higher presence of diabetes mellitus (22% vs. 7.5%, p<0.05) and dyslipidemia (66.7% vs. 36.9%, p<0.05). The patients with a TAD showed a lower presence of dyslipidemia (28% vs. 50%, p<0.05) comparing to patients with no TAD.

**Table 1:** Presence of hypertension, diabetes mellitus, dyslipidemia, family history of the aortic disease, connective tissue disease and previous inflammatory process in patients with different aortic pathologies

		**Hypertension**	**Diabetes mellitus**	**Dyslipidemia**	**Family history of the aortic disease**	**Connective tissue disease**	**Inflamatory process**
Aneurysms	thoracic	49 (75%)	4 (6%)	30 (46%)	1 (1,5%)	3 (5%)	4 (6,2%)
	abdominal	17 (94,4%)	4 (22%)**	12 (66,6%)**	0	0	3 (17%)
both thoracic and abdominal		66 (80%)	8 (10%)	42 (50%)	1 (1%)	3 (4%)	7 (8,4%)
Dissections		54 (79,4%)	6 (8,8%)	19 (28%)**	2 (3%)	3 (4,4%)	6 (8,8%)
**All cases**		**120 (80%)**	**14 (9,3%)**	**61 (40%)**	**3 (2%)**	**6 (4%)**	**13 (9%)**

** - p<0.05, other cases p>0.05.

**Table 2:** Presence of hypertension, diabetes mellitus, dyslipidemia, family history of the aortic disease, connective tissue disease and previous inflammatory process in patients with different histopathological diagnoses

	**Hypertension**	**Diabetes mellitus**	**Dyslipidemia**	**Family history of the aortic disease**	**Connective tissue disease**	**Inflamatory process**
**Low grade atherosclerosis**	16 (76%)	3 (14,2%)	8 (38%)	0	1 (4,8%)	1 (4,8%)
**High grade atherosclerosis**	44 (88%)	6 (12%)	23 (46%)	0	0	7 (14%)
**Low grade medial degeneration**	25 (73,5%)	5 (14,7%)	13 (38,2%)	2 (5,9%)	1 (2,9%)	2 (5,9%)
**High grade medial degeneration**	54 (75%)	4 (5,6%)	28 (38,9%)	1 (13,9%)	5 (7%)	5 (7%)

In all cases p>0.05.

**Table 3:** Presence of hypertension, diabetes mellitus, dyslipidemia, family history of the aortic disease, connective tissue disease and previous inflammatory process in different age groups

		**Hypertension**	**Diabetes mellitus**	**Dyslipidemia**	**Family history of an aortic disease**	**Connective tissue disease**	**Inflamatory process**
Thoracic aneurysms	≥65a	22 (88%)	1 (4%)	14 (56%)	0	0	1 (4%)
	<65a	27 (67,5%)	3 (7,5%)	16 (40%)	1 (2,5%)	3 (7,5%)	3 (7,5%)
Dissections	≥65a	19 (90,5%)	3 (14,3%)	6 (28,6%)	0	0	1 (4,8%)
	<65a	35 (74,4%)	3 (6,4%)	13 (27,7%)	2 (4,3%)	3 (6,4%)	5 (10,6%)

In all cases p>0.05.

### Medial degeneration

Medial degeneration was diagnosed in 106 (70%) patients including 55 (52%) aneurysms and 51 (48%) TADs. High grade medial degeneration was found in 50% of patients with thoracic aortic aneurysms < 65 years of age vs. 44% in patients ≥ 65 years of age (p = 0.64) and in 36% of patients with aortic dissections < 65 years of age vs. 14% in patients ≥ 65 years of age (p = 0.07).

### Atherosclerosis

Atherosclerosis was diagnosed in 71 (47%) patients including 46 (65%) aortic aneurysms and 25 (35%) aortic dissections. All specimens represented Type V atherosclerosis according to the AHA classification [10]. High grade atherosclerosis was found in 23% of patients with thoracic aneurysms < 65 years of age vs. 36% in patients ≥ 65 years of age (p = 0.24) and in 13% of patients with aortic dissections < 65 years of age vs. 52% in patients ≥ 65 years of age (p< 0.001).

### Aortitis

**Figure 6:**
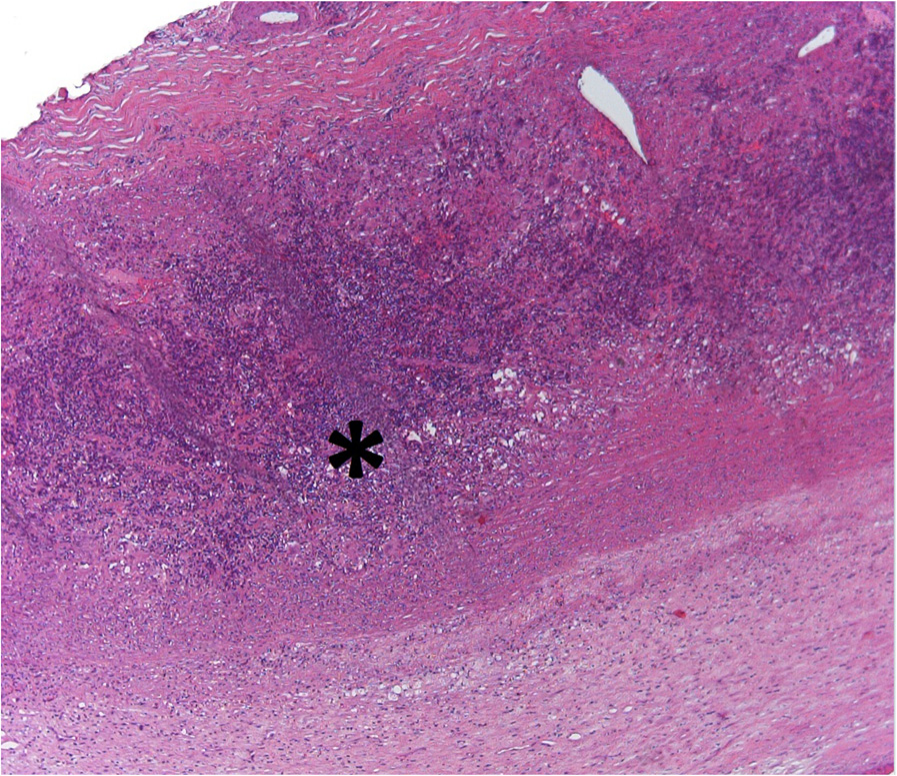
**Aortic wall segment of the patient with giant cell arteritis.** In this form of vasculitis giant cell infiltrates were observed within tunica media (*). [Hematoxylin-eosin staining, original magnification 200×].

**Figure 7:**
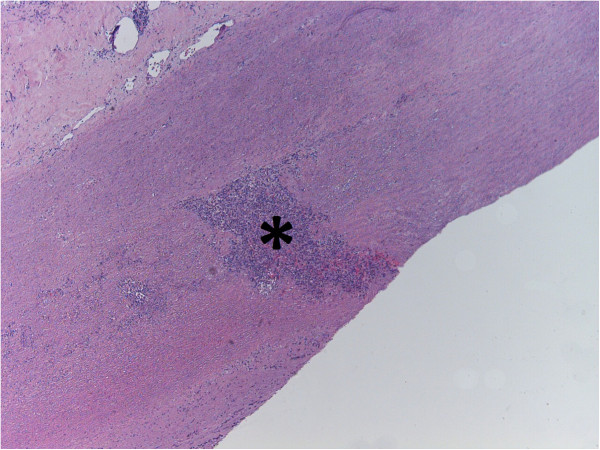
Focal plasma cell infiltration (*) in a patient with a complex chronic Type A aortic dissection. [Hematoxylin-eosin staining, original magnification 200×].

There were only two cases of aortitis in this series. A giant cell infiltration was diagnosed in a patient with an aneruysm of the aorta ascendens (Figure 6). The second patient showed plasma cell infiltration with clinical diagnosis of a complex chronic type A aortic dissection (Figure 7).

### Sex differences

There were 45 (30%) women in this series. Women were older than men (62 ± 15 years vs. 57 ± 14 years, p = 0.03). Distribution patterns of acute and chronic thoracic aortic pathologies were similar. Regarding infrarenal aneurysms, the incidence of male patients prevailed (n = 17, 16% vs. n = 1, 2%, p = 0.02).

### Discussion

Medial degeneration was the most frequent diagnosis in this series of aortic specimens. Medial degeneration was equally common in patients above and below 65 years of age. However, in cases with acute type A aortic dissections, high grade atherosclerosis was the leading histopathological diagnosis in patients older than 65 years. Acute type A aortic dissections seem to have different underlying pathologies in different age groups.

This series represents a one-year volume of an aortic referral center. The intention of this analysis was to correlate histopathological to morphological findings from imaging as well as to raise our intraoperative macroscopic and subjective impressions to a microscopic and objective level.

According to the risk factors a height prevalence of hypertension was observed in all of the patient groups. Especially the TAD group ≥ 65 years of age showed a relatively high rate of hypertension with no clinical diagnosis of connective tissue disease in this group. Generally, patients with an aortic dissection showed a lower presence of dyslipidemia. A significantly higher rate of diabetes mellitus, dyslipidemia and inflammation was noticed in the patients with an infrarenal aneurysm. This fact strongly correlates with the high prevalence of high grade atherosclerosis in this group.

Interestingly, medial degeneration was a very common finding in aneurysms as well as in acute type A aortic dissections. From literature, tradition and terminology one would expect a high incidence of atherosclerosis in chronic ascending aortic aneurysms [9,10]. As this was not the case, it seems likely that other factors such as the presence of a bicuspid aortic valve and consecutively altered hemodynamics through the valve may well be a leading mechanism of aneurysm formation [11]. In acute type A aortic dissection one would expect a certain percentage of patients with connective tissue disease which was also not the case in this series [12,13]. These findings do further substantiate the need for accurate and early determination of patients being at risk for acute type A aortic dissection.

In fact the road to anticipation of patients being at risk for acute type A aortic dissection is long and several approaches have been made to define subgroups at risk. Without doubt, patients with bicuspid aortic valves tend to have a faster diameter progression than patients with bicuspid aortic valves. As a consequence the threshold for indicating surgery should be lowered in this subgroup. Furthermore any kind of anuloaortic ectasia or phenotypic association with connective tissue disease such as a bifid uvula in Loeys-Dietz syndrome should alert for close observation and intervention in case of progression [14]. Finally, functional imaging may lead the way to a better anticipation of aortic events [15].

To our surprise, atherosclerosis was a common finding in acute type A aortic dissection. This finding warrants further discussion. Connective tissue disease as well as hypertension are regarded as the most common risk factors for acute type A aortic dissection whereas atherosclerosis per se is not being associated with acute type A aortic dissection [16,17]. However, it seems most likely that atherosclerosis does not only always have an obliterative component but may also be responsible for dilatative components as well. These results have been confirmed by others and can also be observed in our findings [18,19]. This also explains the low incidence of coronary artery disease in patients with acute type A aortic dissection.

Women were older than men in this series which is not surprising due to the known higher mean age of women being affected by cardiovascular disease [20]. However, distribution patterns of acute and chronic aortic pathology were similar. Furthermore, the incidence of medial degeneration as well as atherosclerosis was comparable. As can be read in the corresponding literature the exception was seen in infrarenal aneurysms which show a clear male prevalence [21,22].

## Conclusions

Medial degeneration was the most frequent diagnosis in this series of aortic specimens. Medial degeneration was equally common in patients above and below 65 years of age. However, type A aortic dissections seem to have different underlying pathologies according to different age groups. In patients below 65 years of age there was a clear correlation between medial degeneration and type A aortic dissections whereas in patients above 65 years of age, type A aortic dissections were associated with atherosclerosis. The incidence of aortitis in this series was negligibly low which is undoubtedly related to ethnical as well as geographical factors [23].

### Limitations of the study

This study entails all limitations of a single center observational report. In particular, distribution patterns of acute and chronic aortic pathology may well be a regional phenomenon as we have observed and may not be valid in another setting.

## Abbreviations

HE: Hematoxylin-eosin staining; AHA: American Heart Association; TAD: Thoracic aortic dissections; TAA: Thoracic aortic aneurysms; AAA: Abdominal aortic aneuryms.

## Competing interests

The authors declare that they have no competing interests.

## Authors’ contributions

AJ carried out the study design, collected the clinical data of the patients drafted the manuscript and participated in the interpretation of the histopathological speciemens. GB carried out the interpretation of the histopathological speciemens. TD participated in the study design and performer the statistical analysis. AK participated in the collection of the clinical data and interpretation of the results. GL participated in the study design and coordination. ME carried out the interpretation of the results, participated in the study design and helped to draft the manuscript. All authors read and approved the final manuscript.
